# Cardiovascular Risk Stratification: From Phenotype to Genotype?

**DOI:** 10.5935/abc.20180010

**Published:** 2018-01

**Authors:** Marcio Sommer Bittencourt

**Affiliations:** Hospital Israelita Albert Einstein e Faculdade Israelita de Ciências da Saúde Albert Einstein - São Paulo, SP - Brazil; Centro de Pesquisa Clínica e Epidemiológica - Hospital Universitário e Instituto do Câncer do Estado de São Paulo (ICESP) - Universidade de São Paulo - São Paulo, SP - Brazil; Diagnósticos da América (DASA) - São Paulo, SP - Brazil

**Keywords:** Acute Coronary Syndrome/genetic, Metabolic Syndrome, Risk Factors, Risk Assessment, Polymorphism, Genetic

Cardiovascular risk scores, such as the Framingham score, have been strongly recommended by clinical guidelines on the assessment of cardiovascular risk.^1^ However, several studies have shown limitations for their use,^2,3^ particularly in patients at intermediate risk, young patients with a definite family history, and women. Among different tools aimed at improving risk stratification by complementary methods, the use of genetic information has been proposed to enhance risk prediction.^4^

Although many genetic polymorphisms have been associated with increased cardiovascular risk, the additional value of their use in the clinical practice has not been defined yet. One of the reason for such limitation lies on the fact that atherosclerosis is a multifactorial disease, and the individual role of each polymorphism is limited. Since many polymorphisms associated with atherosclerotic disease have been identified, some authors have investigated combinations of several polymorphisms aiming to develop genetic scores that serve as stronger predictors of cardiovascular risk. Nevertheless, despite great enthusiasm about the role of genetic information on the development of cardiovascular risk, previous data have suggested that even with the combination of more than 50 polymorphisms, the best risk stratification achieved was still poor, and of low clinical value in its current form.^5^

In another attempt to assess the role of genetic scores on atherosclerotic disease, Fisher et al. investigated 116 individuals with metabolic syndrome and recent history of acute coronary syndrome (ACS) to assess the association between several genetic polymorphisms and the extension of coronary artery disease (CAD).^6^ While lipoprotein lipase gene polymorphism was associated with atherosclerotic load, polymorphism-derived genetic score was not associated with atherosclerotic load defined by Gensini score in invasive angiography.

These findings may be explained by several reasons. First, the sample size was relatively small for a genetic study. Second, the value of each polymorphism, alone is usually small. In addition, while most studies use gene panels composed of tens of markers, only seven markers were used in this study. Finally, the population studied was different from those of population-based studies. Using recent ACS as an inclusion criterion, the present study included not only patients with clear evidence of atherosclerosis, but also with recent history of plaque instability. The selection of individuals with such different phenotypes may also have affected the development of a genetic score.

Despite these limitations, the study expands the literature on genetic assessment of CAD, demonstrating once again that this association is not simple.

In order to make genetic score part of routine clinical care, improvement of genetic sequencing techniques, development of studies involving larger, representative populations, and the use of modern data modeling methodologies that incorporate nuances beyond the linear association between predictors and outcomes are required.^7^
